# 3D Printed Biomodels for Flow Visualization in Stenotic Vessels: An Experimental and Numerical Study

**DOI:** 10.3390/mi11060549

**Published:** 2020-05-29

**Authors:** Violeta Carvalho, Nelson Rodrigues, Ricardo Ribeiro, Pedro F. Costa, Rui A. Lima, Senhorinha F.C.F. Teixeira

**Affiliations:** 1 ALGORITMI Center (CAlg), University of Minho, 4800-058 Guimarães, Portugal; a78923@alunos.uminho.pt (V.C.); nelson.rodrigues@dps.uminho.pt (N.R.); 2 Mechanical Engineering and Resource Sustainability Center (MEtRiCS), University of Minho, 4800-058 Guimarães, Portugal; 3 BIOFABICS, Rua Alfredo Allen 455, 4200-135 Porto, Portugal; ricardo.ribeiro@biofabics.com; 4 Transport Phenomena Research Center, Department of Chemical Engineering (CEFT), Engineering Faculty, University of Porto, 4200-465 Porto, Portugal

**Keywords:** atherosclerosis, in vitro biomodels, CFD, 3D printing, stereolithography, blood flow, blood analogues, hemodynamics

## Abstract

Atherosclerosis is one of the most serious and common forms of cardiovascular disease and a major cause of death and disability worldwide. It is a multifactorial and complex disease that promoted several hemodynamic studies. Although in vivo studies more accurately represent the physiological conditions, in vitro experiments more reliably control several physiological variables and most adequately validate numerical flow studies. Here, a hemodynamic study in idealized stenotic and healthy coronary arteries is presented by applying both numerical and in vitro approaches through computational fluid dynamics simulations and a high-speed video microscopy technique, respectively. By means of stereolithography 3D printing technology, biomodels with three different resolutions were used to perform experimental flow studies. The results showed that the biomodel printed with a resolution of 50 μm was able to most accurately visualize flow due to its lowest roughness values (Ra = 1.8 μm). The flow experimental results showed a qualitatively good agreement with the blood flow numerical data, providing a clear observation of recirculation regions when the diameter reduction reached 60%.

## 1. Introduction

Atherosclerosis is considered the pathological basis of several cardiovascular diseases and the leading cause of death worldwide, with over 20 million deaths every year. It is a silent and progressive disorder characterized by abnormal lipid deposition within the intima and commonly affects large- and medium-sized arteries, like coronary arteries [1,2]. 

Given the clinical importance of this disease worldwide, hemodynamics research has increased exponentially. In vivo experimental methods provide the most realistic physiological data and remain essential for establishing an adequate understanding of the basic biophysical phenomena that happen in vivo. However, even performing these studies in animal models, neither human physiology nor human disease is fully recapitulated. In these types of experiments, the measurements are often inaccurate, and the experiments are expensive, have low reproducibility, and have associated ethical issues [3,4,5,6]. Therefore, engineered in vitro biomodels have gained widespread attention and have been developed to overcome those limitations. These methods are often used to validate numerical studies and to complement and confirm results from in vivo experiments [7]. Numerical hemodynamic studies can act as an auxiliary tool to investigate such arterial diseases, offering advantages over experimental analysis since they are faster. The construction of more realistic virtual models has become more flexible and they reduce the lead times and costs of new designs and developments. Although experimental and numerical methods are important by themselves, these studies should be compared to verify the accuracy of the results and thus validated [8,9]. 

The flow dynamics of stenotic arteries have been subjected to extensive studies with early in vitro [10,11] and in silico studies [12,13]. The interest in understanding the blood flow behavior in the atherosclerosis condition has been continuously growing due to its serious impact on human life. An important geometric factor to the local hemodynamics is the shape of the stenosis. Some experimental and numerical studies applied idealized stenosis shapes [5,14,15,16]. Other studies used of patient-specific geometries based on medical images [6,17,18,19]. Kefayati et al. numerically and experimentally studied the effect of stenosis severity, plaque eccentricity, ulceration on turbulence intensity [14] and on shear stresses measured in vitro [20]. In another study, the authors analyzed the transitional flow [21] using idealized carotid models. Similar studies were conducted by DiCarlo et al. [22,23]. Costa et al. [6] used a patient-specific geometry of a coronary artery with a shape similar to the one presented here. By combining stereolithography (SLA) 3D printing and computed tomography angiography (CTA) data, they obtained a 3D biomodel, and performed both in vitro and in silico studies of the flow through healthy and stenotic arteries. Recently, Stepniak et al. [24] studied the lipid-rich plaque in stenotic coronary artery phantoms using three different 3D printing technologies (fused deposition modelling, SLA, and PolyJet). 

Despite the variety of methods to produce arterial phantoms for flow visualization, 3D printing technologies have become popular due to its capability to fabricate full 3D structures that closely mimic the shape of human blood vessels [25,26]. There are different 3D printing methods that have been used in this field, such as fused deposition modelling [3,24,27,28,29], Inkjet [30], PolyJet [24,31] and binder jetting [27]. In addition to these methods, a suitable technique to fabricate vessel models is SLA, which provides very high printing resolutions and accuracy, well-defined details, slightly visible layer lines, and an extremely smooth surface finish. These characteristics play an important role aiding the visualization process during experimental studies. In this technique, the models are manufactured layer by layer using a photocurable liquid resin that is hardened by applying a low-power ultraviolet (UV) laser, a process called photopolymerization [6,32,33,34]. 

After the fabrication process, in vitro velocity fields of blood flow can be measured using several techniques [18,30,35,36,37,38,39]. However, in the present study, blood flow measurements and visualizations were performed using a high-speed video microscopy system.

In past decades, many in vitro studies have been conducted, however, none of them considered the influence of roughness on the flow visualization. The present paper shows the influence of this parameter on the visualization of tracer particles and the effect of stenosis severity on hemodynamics. Using numerical and experimental approaches, the five degrees of stenosis (DOS) seen in human arteries were evaluated, in addition to a healthy model fabricated by the stereolithography 3D printing technique.

## 2. Materials and Methods

### 2.1. Coronary Artery 3D Biomodels Design and Fabrication

The 3D biomodels were custom-designed using the online platform BIOFABICS TOOLBOX (www.biofabics-toolbox.com) and custom-manufactured using a rigid material (Biofabics, Porto, Portugal) at printing resolutions of 50, 100, and 150 μm. The biomodels were designed with tool number 3 of the BIOFABICS TOOLBOX, employing a 24-well plate layout, a 59 mm total channel length, a 3 mm channel 1 diameter, a 3 mm channel 2 diameter, a 6 mm stenosis length, a 0.5 mm base thickness, and a total device height of 5.5 mm. The DOS selected for this study were 0%, 50%, 60%, 70%, and 80% to capture the changes in flow behavior when the severity of the stenosis increases.

**Figure 1:**
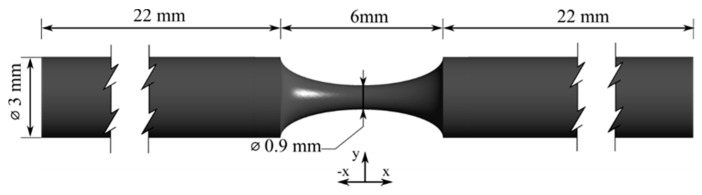
Dimensions of the 70% stenotic model.

The shape of the artery was a full cylindrical tube with a circular cross-section and an axisymmetric blockage (Figure 1). The selected diameter (3 mm) was based on the literature [40,41] and the length of the stenosis was defined as twice the normal diameter, an assumption applied by several researchers [42,43,44,45]. In addition, the length of the inlet zone (22 mm) was selected to ensure the creation of a fully developed inlet flow.

The DOS is estimated by comparing the minimum diameter of the vessel at the lesion site ($d_{stenosis})$ to the diameter of the straight section ($d_{straight}$) without any stenosis, Equation (1). It can be defined as minimal, moderate, or severe: minimal if the narrowing was less than 50%, moderate if between 50% and 70%, and severe or significant for a diameter reduction of 70% or more [46].

(1) $$stenosis\;\left(\%\right)=\left(1-\frac{d_{stenosis}}{d_{straight}}\right)\times 100$$

### 2.2. Blood Analogue Fluid and Flow Rate

Recently, the use of blood analogues has been increasing due to the difficulties associated with the manipulation of real blood in vitro because of ethical, economical, or hazardous issues [47,48,49]. 

In this study, the fluid selected as the blood analogue was a dimethyl sulfoxide (DMSO)/water mixture (52%/48% *w*/*w*) with properties similar to blood μ=0.003153426 Pa·s and $\rho =1072.033\;\mathrm{kg}\cdot m^{-3}$), where μ is the viscosity and ρ is the density of the fluid. The density of the biofluid was measured using a density meter (DMA 5000M, Anton Paar^®^, Graz, Austria) [50]. The refractive index of this mixture matches the refractive index of the biomodels, eliminating the visual distortion produced by the curvature of the walls. By using a syringe pump, the infusion flow rate was kept constant at 5 mL/min. The same flow rate was used to perform numerical simulations.

### 2.3. Experimental Setup for Roughness Measurements and Flow Characterization

**Figure 2:**
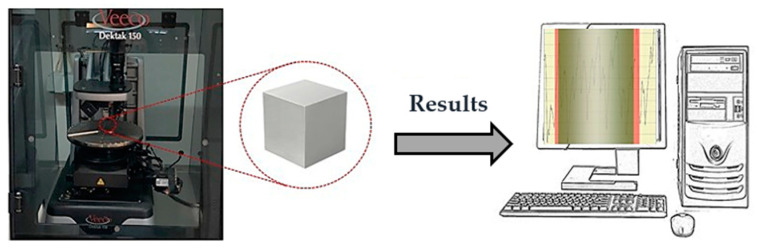
Experimental setup for roughness measurements.

To understand the influence of the print resolution in flow visualization, roughness measurements were performed using a surface profiler (Dektak^®^ 150, Veeco, Plainview, NY, USA) (Figure 2). Considering the difficulty and inaccuracy of the roughness measurements on circular surfaces, cubes printed with the respective resolutions were used to make the comparison. 

**Figure 3:**
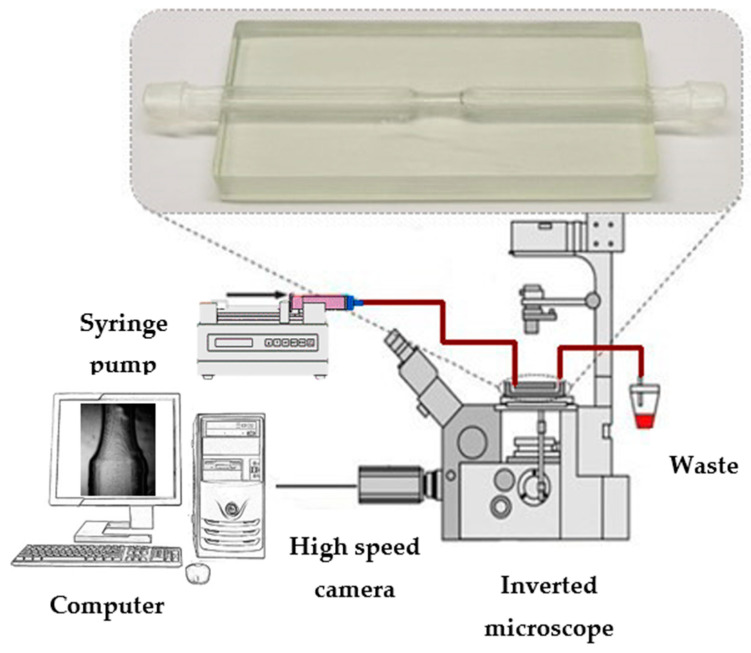
Experimental equipment used to control and visualize the flow of the blood analogues.

In Figure 3, the experimental setup used to perform the flow visualization is represented. This was composed of an inverted microscope (IX71, OLYMPUS, Tokyo, Japan) combined with a high-speed video camera (Fastcam SA3, Photron, San Diego, CA, USA) and the objective lens used in this study had a magnification of 2.5×. To control and impose steady flow rates through the channels of the biomodel, a syringe pump (LEGATO^®^ 100, Holliston, MA, USA) was used. All images were captured and recorded by a high-speed video camera (Fastcam SA3, Photron, USA). The microparticles used in suspension of the blood analogue fluid were 40 μm monosized spherical tracer particles (CA 40, Spheromers^®^) at a concentration of 0.2%. 

### 2.4. Numerical Approach

Numerical simulations were performed in the CFD package ANSYS Fluent (version R2), which uses the finite volume methodology. 

**Figure 4:**
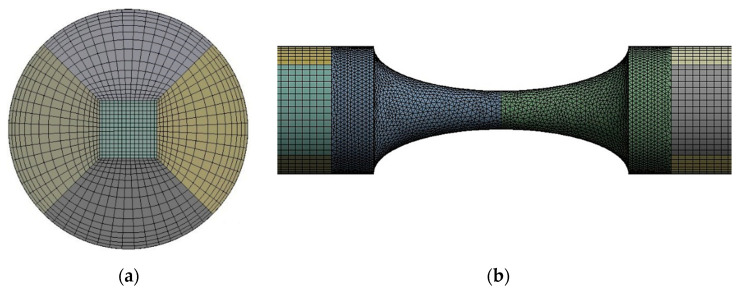
Hybrid mesh generated for the 70% model: (**a**) inlet view; (**b**) front view.

For each model, a hybrid mesh was generated with tetrahedral cells with inflation layers in the stenosis zone and hexagonal cells with a bias factor in the remaining geometry. The inflation and bias factor were applied to improve the accuracy of the computed wall shear stresses (WSS). Several mesh tests were performed to guarantee a grid-independent solution. The mesh that combines accuracy and lower computation time for the 70% stenosis case has 498,974 elements (Figure 4). For the remaining models, similar meshes were performed.

Although in small arteries and capillaries the blood exhibits non-Newtonian behavior in vessels with a diameter superior to 1 mm, in the coronary arteries, non-Newtonian behavior can be neglected due to the predominant high-shear rates [51,52,53,54]. In this study, the blood was considered as an incompressible, homogeneous, and Newtonian fluid. The no-slip boundary condition was applied at the artery walls, which were assumed as rigid and motionless. The outlet gauge pressure was set to zero and there was no backflow. Although the blood flow in the coronary arteries is time-dependent, the current investigation first simplifies it to a steady flow condition, with constant inputs, assuming a laminar regime. This allows for validation with the experimental data.

Under these assumptions, three-dimensional blood flow through an artery with stenosis can be described by the steady continuity and Navier–Stokes equations, Equations (2) and (3):

(2) $$\nabla \cdot \vec{u}=0$$

(3) $$\rho \left(\frac{\partial \vec{u}}{\partial t}+\vec{u}\cdot \nabla \right)=-\nabla p+\mu \nabla^{2}\vec{u}$$

where $\vec{u}$ is the velocity vector, p is the static pressure, ρ is the fluid density, and μ is the dynamic viscosity. 

These governing equations were solved by the semi-implicit method for pressure linked equations (SIMPLE) scheme [8].

### 2.5. Image Processing

The images were obtained essentially in the post stenotic section of the biomodel by using a frame rate of 1000 frames/second. All sets of frames were analyzed using the Z Project method in ImageJ software (Bethesda, MD, USA) [55]. The number of analyzed frames was about 800 for each movie and the recorded sequence of images was replaced by an image that represents the minimum or maximum intensity in each pixel. By using this methodology, it is possible to observe clearly the flow behavior.

To perform the particles’ velocity measurements, the manual MtrackJ plugin was used. This is an ImageJ plugin that allows the tracking of moving particles and to calculate their velocities automatically. 

To estimate the difference between the experimental results and the numerical values, Equation (4) was used:

(4) $$\%\;error=\frac{\left|Experimental\;value-Numerical\;value\right|}{Numerical\;value}\times 100$$

## 3. Results and Discussion 

### 3.1. Printing Resolution Effect

**Figure 5:**
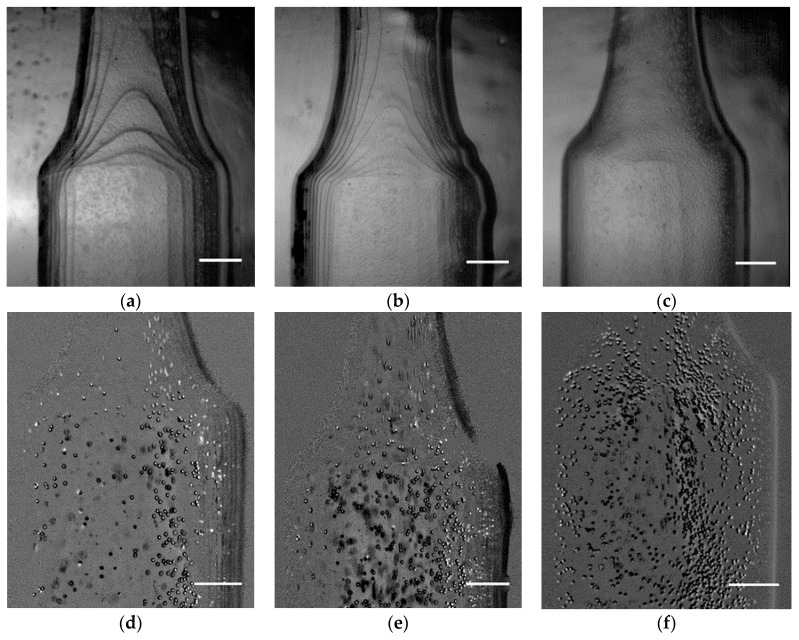
Images obtained for each printing resolution: (**a**) 150 μm; (**b**) 100 μm; (**c**) 50 μm and the respective images with tracer particles obtained by the high-speed camera: (**d**) 150 μm; (**e**) 100 μm; (**f**) 50 μm. Scale bar, 1 mm.

To understand the influence of the print resolution in flow visualization, three different cases were tested (50, 100, and 150 μm). Initially, it was analyzed in a qualitatively, observing the flow through a microscope. As can be seen in Figure 4, there was a significant improvement when the print resolution was reduced to 50 µm (Figure 5c). In Figure 5a,b, there are accentuated lines that affect the flow visualization as well as particle tracking. 

To better understand the impact of the printing resolution in the assessment of the experimental results, additional images from ImageJ were obtained (Figure 5d–f). Those images were recorded before the application of the Z Project tool. 

Through the analysis of the results, it was possible to observe the difficulty to track the tracer particles near the walls for the printing resolutions of 100 and 150 μm (Figure 5d,e). Between these two biomodels, there was almost no difference. However, in comparison to the 50 μm biomodel, the differences were quite significant. For instance, by using images obtained with the 50 μm biomodel (Figure 5f), the tracer particles could be tracked without almost any influence of the channel wall. In the remaining cases, the images were too noisy near the walls, which promoted the generation of large errors when tracking those particles. These results were extremely relevant in flow experiments as it was essential to obtain high-quality flow images near the walls to obtain accurate in vitro results, namely in WSS and velocity measurements. 

**Figure 6:**
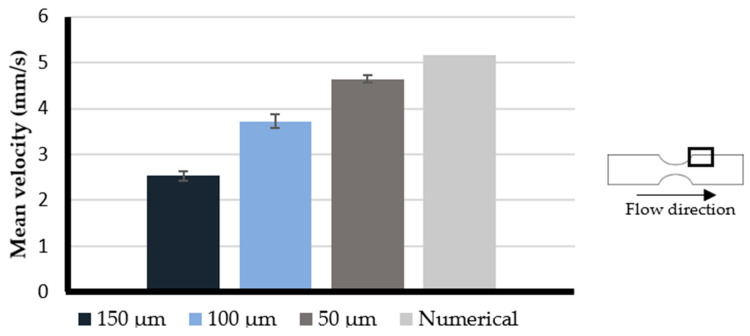
Comparison between the mean velocities measured experimentally near the wall at each model with the mean velocity estimated numerically. The experimental measurements are expressed as the mean ± standard deviation according to a *t*-test analysis at 95% confidence interval.

Following the previous results, the effect of the printing resolution in the experimental measurements near the wall was quantitatively evaluated, where the results were most affected by this parameter. For this purpose, the mean particles’ velocity was measured experimentally and numerically near the wall in the post stenotic section of the 50% stenotic model. The experimental measurements were performed at an approximate distance of 100, 105, and 145 µm from the wall. Those distances corresponded to the 50, 100, and 150 µm models, respectively. Numerically, the velocity measurements were obtained from a plane located and with an area close to which the experimental measurements were made. When analyzing the results depicted in Figure 6, the model with values closest to the numerical results is the 50 µm model. The 150 µm model presented the worst results; the velocities measured experimentally were much lower than those predicted numerically. The 100 µm model presented results between the two models mentioned above, but it was significatively better than the 150 µm model. 

The error associated with the measurements was also calculated and, as expected, the measurements in the 50 µm model presented the lowest error of about 10%. The measurements in the 100 and 150 µm models presented higher errors, approximately 28% and 50%, respectively. Considering these results, we concluded that the 50 and 100 µm models are the most adequate to perform the experimental studies with more reliability and the 50 µm model produced the most accurate results. 

Through the analysis of these results, we verified the direct influence of printing resolution on the quality and reliability of particles’ velocity measurements near the wall. 

Additionally, roughness measurements were recorded to verify the previous observations. The main roughness parameter was the average roughness (Ra). This parameter evaluates the average standard deviation of the heights (peaks and valleys) in a scanned profile to compute the degree of roughness [56,57]. 

**Figure 7:**
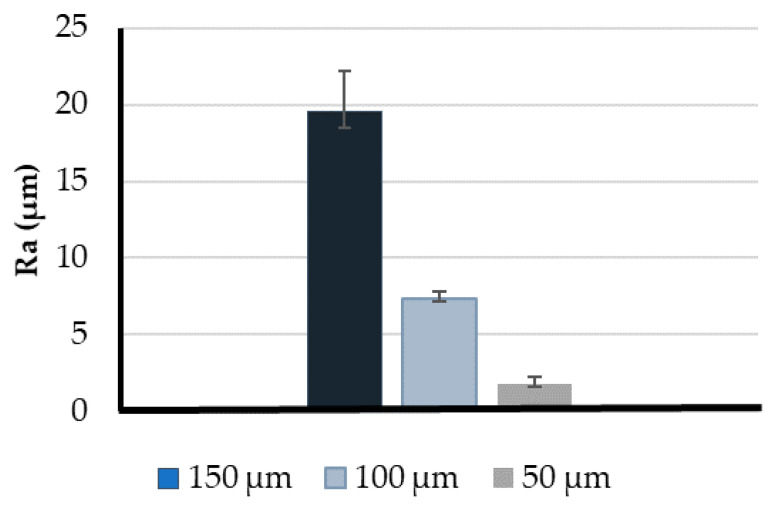
Comparison between the roughness parameter, Ra, at three different print resolutions. The measurements are expressed as the mean ± standard deviation according to a *t*-test analysis at 95% confidence interval.

To perform the roughness tests, different cubes were printed with the three different resolutions (three measurements for each case). Figure 7 shows the average Ra values obtained per each resolution proving that this parameter influences the models’ roughness. The higher value was measured in the 150 μm model with an average Ra of approximately 19.53 μm. In turn, the 100 μm model had a significantly lower roughness (7.35 μm). However, the best model was the one printed with the lowest resolution (50 μm), presenting the lowest roughness values with an average Ra of 1.78 μm. 

From the results presented in this section, we concluded that with a printing resolution of 50 μm, the experimental results are more accurate and the lowest surface roughness can be achieved. Therefore, the remaining tests were performed in those models.

### 3.2. Velocity Profiles and Wall Shear Stress Evaluation

**Figure 8:**
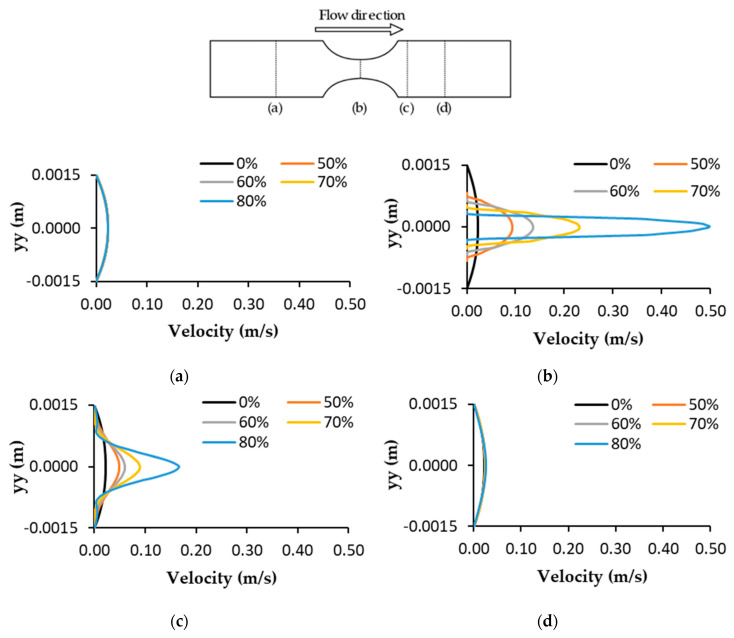
Comparison of velocity profiles at different axial positions for the different degrees of stenosis: (**a**) before stenosis, (**b**) stenosis throat, (**c**,**d**) after stenosis.

The numerical prediction of the effect of stenosis severity on blood velocity profiles at different sites in the artery for different DOS is depicted in Figure 8. Figure 8a shows that the velocity profile was fully developed at the beginning of the channel and was not significantly affected by the DOS. It was seen only as a slight difference in the maximum velocity between the healthy model and the stenotic cases. Figure 8b shows that at the stenosis throat for all DOS, the velocity increased, reaching its maximum. As the throat diameter reduced, the velocity increased and the highest speed was achieved with a DOS of 80% (0.50 m/s). Figure 8c,d show the corresponding velocity profiles at two different locations in the downstream region of stenosis. In Figure 8c, the velocity profiles are a little disturbed and fluctuating due to the presence of secondary recirculating flows for some models. The remaining velocity profiles did not display that behavior, showing absence or reduced recirculation. In Figure 8d, the flow was stabilized again, although in the DOS of 80%, the velocity was slightly higher.

Table 1 shows the Reynolds number (Re) (ρUD/μ) in the stenotic region for each degree of stenosis, where ρ is the fluid density, U is the maximum velocity in the stenosis throat, D is the stenotic diameter, and µ is the fluid viscosity. As the stenosis severity increased, the Re also increased, which was explained by the velocity increase in that region. Additionally, the maximum Re was approximately 102. Therefore, the laminar flow assumption was adequate in the present simulation.

WSS plays an important role in the development of various vascular disorders, including atherosclerosis [58]. This was a key parameter in the hemodynamic analysis due to its direct relationship to the development of cardiovascular disease and atherosclerosis in general. High WSS was indicated as a contributor to the rupture of the atherosclerotic plaque in human coronary arteries [59] and low WSS was associated with plaque progression in diseased human coronary arteries [60]. For a Newtonian fluid, the applied shear stress ($\tau_{w}$) is proportional to the resulting deformation rate ($\dot{\gamma }$) and the viscosity is constant:

(5) $$\tau_{w}=\mu \frac{\partial u}{\partial y}=\mu \dot{\gamma }$$

**Figure 9:**
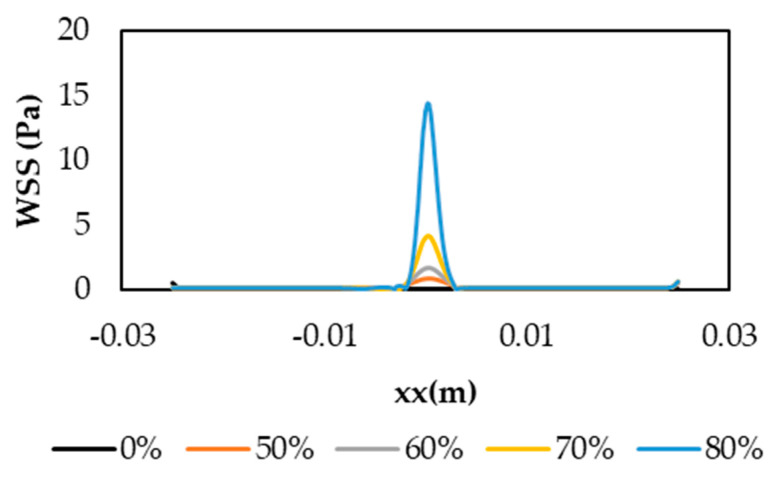
Wall Shear Stresses (WSS) distribution along the vessel wall.

The effect of stenosis on WSS is depicted in Figure 9. The maximum WSS was observed at the throat stenosis and its value increased with the degree of stenosis. This happened because the artery diameter reduction caused higher velocities in the stenosis throat and, consequently, the WSS also increased. Similarly to the previous results, it could be verified that the maximum WSS was reached for a DOS of 80%, being approximately 14.64 Pa. For the healthy model, WSS was almost constant at the value of 0.10 Pa. For the 50%, 60%, and 70% of stenosis, WSS values of 0.86, 1.73, and 4.19 Pa were observed at the throat, respectively. In addition, we noticed that a sudden increase in the WSS from DOS 70% to 80%, showing that the second case was already extreme, possibly leading to acute myocardial infarction. 

### 3.3. Velocity Fields and Flow Visualizations

**Figure 10:**
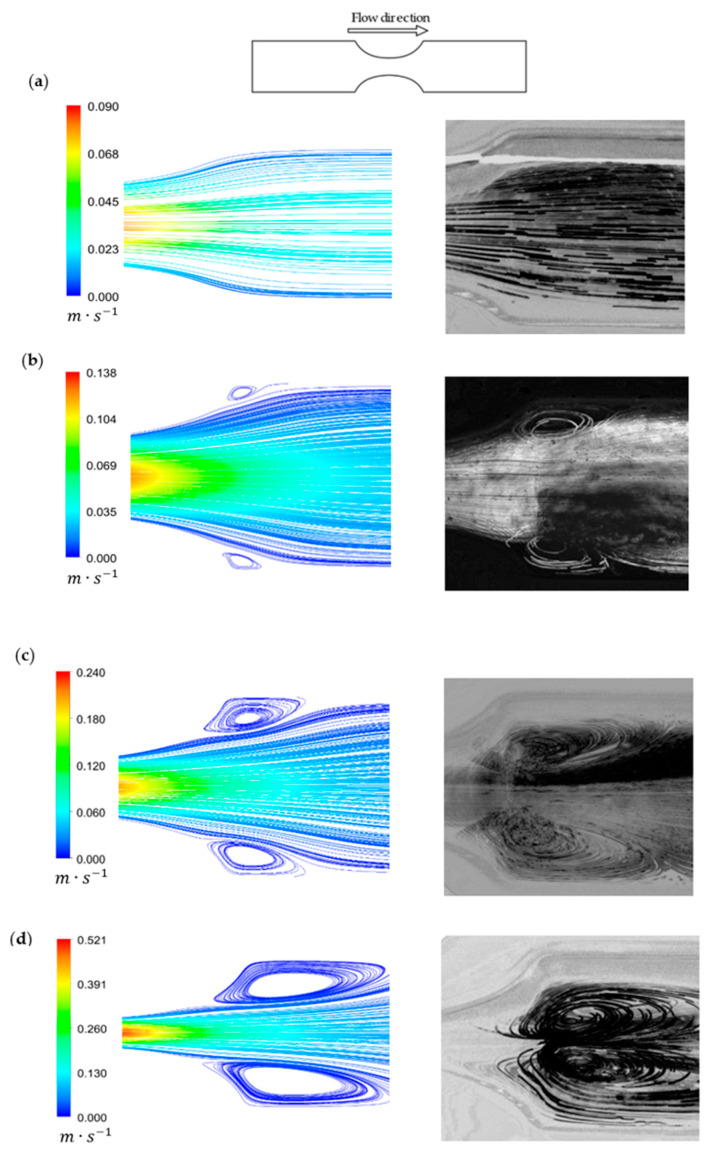
Streamlines for different degrees of stenosis: (**a**) 50%, (**b**) 60%, (**c**) 70%, and (**d**) 80%. Left: Numerical velocity streamlines; Right: Z Project streamlines.

To evaluate the flow behavior, the streamlines and velocity magnitudes were obtained by CFD simulations and the respective Z Project experimental images were compared and they are depicted in Figure 10. 

In general, the numerical data showed qualitatively good agreement with the experimental results. In Figure 10a, the numerical data accurately describe the experimental results, showing that with a 50% degree of stenosis, the fluid flows in a laminar and regular way. Looking at Figure 10b, small recirculation regions appeared and both results were in close agreement. However, for this geometry and also in the remaining stenotic cases, the numerical model always predicted smaller recirculation areas. For the 70% stenosis case (Figure 10c), the results were also similar. Additionally, the numerical and experimental recirculation behavior were different when the diameter reduced to 80% (Figure 10d). Although the recirculation length was in good agreement, experimentally the recirculation tended to be formed closer to the stenotic section and around the center of the biomodel, which did not happen numerically.

These qualitative results showed the importance of in vitro studies to complement and validate numerical studies. 

## 4. Conclusions

The effect of the printing resolution on the flow visualization of tracer particles was qualitatively studied. The results indicated that there is a direct effect of this parameter on the experimental flow measurements and the biomodel, with the printing resolution of 50 μm producing the most reliable results. This model produced experimental measurements closest to the numerical results and a lower roughness. Additionally, the effect of coronary stenosis on hemodynamic changes was numerically and experimentally studied and both results were in good agreement. For both cases, when the diameter reduced to 60%, the formation of small recirculations near the walls of the post stenotic section was observed. The velocity profiles and wall shear stress were evaluated, indicating a significant increase of these parameters in the stenosis throat. 

These results provide new insights and additional information about the importance of printing parameters, namely, the resolution on in vitro biomodels. 

## Figures and Tables

**Table 1:** Reynolds number at the stenotic section for the different degrees of stenosis.

Stenosis Degree	Reynolds
0%	23
50%	45
60%	55
70%	71
80%	102
